# Genetic Diversity Analysis of Soybean Collection Using Simple Sequence Repeat Markers

**DOI:** 10.3390/plants12193445

**Published:** 2023-09-30

**Authors:** Alibek Zatybekov, Moldir Yermagambetova, Yuliya Genievskaya, Svetlana Didorenko, Saule Abugalieva

**Affiliations:** 1 Laboratory of Molecular Genetics, Institute of Plant Biology and Biotechnology, Almaty 050040, Kazakhstan; a.zatybekov@ipbb.kz (A.Z.); m.ermagambetova@ipbb.kz (M.Y.); y.genievskaya@ipbb.kz (Y.G.); 2 Department of Oilseed Crop., Kazakh Research Institute of Agriculture and Plant Growing, Almalybak 040909, Kazakhstan; svetl_did@mail.ru

**Keywords:** soybean, plant height, SSR markers, genetic diversity, clusterization, marker-assisted selection

## Abstract

Soybean [*Glycine max* (L.) Merr.] is a nutrient-rich crop that offers a sustainable source of dietary protein and edible oil. Determining the level of genetic diversity and relationships between various genetic resources involved in breeding programs is very important in crop improvement strategies. This study evaluated 100 soybean accessions with diverse origins for 10 important agronomic traits, including plant height (PH), an important plant adaptation-related trait impacting yield, in conditions in southeastern Kazakhstan for 2 years. The comparison of different groups of PH (tall, middle, and short) using a t-test suggested that the group of plants with the tallest PH provided a higher yield (*p* < 0.001) in relatively dry field conditions. The genetic diversity of the accessions was estimated using 25 simple sequence repeat (SSR) markers previously known to be associated with plant height. The results showed a significant variation among different groups of origin for all measured agronomic traits, as well as high genetic diversity, with the PIC (polymorphism information content) varying from 0.140 to 0.732, with an average of 0.524. Nei’s diversity index ranged between 0.152 and 0.747, with an average of 0.526. The principal coordinate analysis (PCoA) of the studied soybean collection showed that Kazakhstan accessions were genetically distant from European, East Asian, and North American cultivars. Twelve out of twenty-five SSR markers demonstrated significant associations with ten studied agronomic traits, including PH (*p* < 0.05). Six SSRs with pleiotropic effects for studied traits were selected, and their haplotypes with phenotypic effects were generated for each soybean accession. The obtained results can be used in soybean improvement programs, including molecular-assisted breeding projects.

## 1. Introduction

Soybean is the world’s largest oilseed crop, accounting for about 60% of global production [1]. Soybean is a good source of protein and vegetable oil that can be used both for human consumption and animal feed. It is a good source of heart-healthy fats, and soymeal is a rich protein source often used to feed livestock, poultry, and aquaculture. Globally, 122 million hectares of soybeans are planted, and the total world production is 341.8 million tons [1]. Brazil is the largest global producer and exporter of soybeans, followed by the United States, Argentina, and China, while Kazakhstan is only the 21st [2].

In Kazakhstan, soybeans are grown mainly in the southeast of the country; however, the government has declared an area expansion program, on the basis of which the soybean acreage should be increased to 1 million ha in the southern, southeastern, eastern, and northern regions of the country [3,4]. In order to achieve better plant adaptation ability, seed production, and quality, it is essential to increase the diversity of local soybean cultivars by introducing genetically distant germplasm. Currently, the use of diverse germplasm from different parts of the world is the priority for the development of soybean genotypes adaptable to different environments and with higher grain yields and improved seed quality.

Flowering time and plant height (PH) are essential agronomic traits that are related to better adaptation to diverse environments and directly influence soybean production and profitability [5,6,7]. Understanding the genetic diversity associated with these traits within the world soybean collection is one of the most important priorities for local breeding programs [6]. Particularly, PH is a very important trait in soybean breeding since it directly correlates with yield [5,6]. Since taller plants produce more productive nodes and increase yield, and too-tall PH causes lodging, it is important to find an optimum range for this quantitative trait [5,6]. Therefore, the combination of advanced molecular tools with traditional breeding techniques is becoming critical for crop improvement [8,9]. These tools can help identify genes for desirable traits based on studies of diverse germplasm and select donors with advantageous allele combinations. At the same time, traditional breeding methods may apply these tools to construct new cultivars with higher yield potential. Therefore, assessing genetic diversity in soybeans is an efficient approach for selecting promising genotypes. This can be achieved by combining morphological and molecular markers to identify individuals with the desired traits and genetic diversity. Previously, the collection of 120 accessions from different parts of the world, including 18 accessions from Kazakhstan, was genetically evaluated using four major maturity genes (*E1*, *E2*, *E3*, and *E4*) that control flowering time [10]. However, in Kazakhstan, the assessment of genetic factors for PH has not yet been properly addressed. Worldwide, several publications indicate a strong genetic heritability of PH and have identified responsible genes and QTLs (quantitative trait loci) in soybean [11,12,13,14]. For instance, Xue and colleagues (2019) determined 36 QTLs controlling PH in multiple developmental stages [11]. Yang and co-workers (2021) identified 19 loci containing 51 QTLs for PH across four environments [12]. Wang and colleagues (2022) determined two candidate genes (*Glyma.02G133000* and *Glyma.05G240600*) involving plant height using studies in multiple environments and backgrounds [13]. Chen and co-authors (2020) [14] established that under short-day (SD) conditions, the gmap1 quadruple mutant exhibited delayed flowering and increased node number and internode length, resulting in taller plants than the wild type. Conversely, the overexpression of *GmAP1a* resulted in early flowering and reduced plant height compared to the wild type under SD conditions [14].

One of the ways to assess the genetic background behind this trait is through the application of informative types of DNA markers, including simple sequence repeats (SSR, also known as microsatellites) and single nucleotide polymorphism (SNP) markers. In the past decades, SSR and SNP markers have been widely used to study genetic diversity [15,16,17,18] and search for associations between markers and traits [19,20]. Both SSRs and SNPs are ubiquitous in the genome of most crops and, therefore, potentially useful to determine the genetic structure of a population and study the evolutionary history and phylogenetic relationships of species. Nevertheless, SSRs tend to have a higher genetic variation level than SNPs [21,22,23]. SSRs are based on variations in the number of repeats in short DNA sequences, which can be highly polymorphic and may have a larger number of alleles per locus. This variability makes SSR markers suitable for studying diverse populations, detecting fine-scale genetic differences, and characterizing genetic diversity. SSRs are generally codominant, meaning both alleles at a marker locus can be detected separately [23,24]. This allows for the precise genotyping and identification of heterozygotes. The genotyping process for SSRs can be less expensive than high-throughput SNP genotyping methods, which often require sophisticated equipment and analysis pipelines [18,22]. Therefore, among the various types of molecular markers, SSRs have emerged as a powerful tool for determining genetic diversity in plants.

Several studies have confirmed that SSR markers are a convenient tool to identify genetically diverse soybean breeding materials and broaden the genetic background of available germplasm [25]. The genetic diversity associated with PH is particularly interesting because it influences various agronomic traits, including lodging resistance, light interception, and nutrient utilization [12,25,26]. Additionally, identifying SSR markers associated with PH and yield can contribute to the development of high-yielding soybean cultivars by facilitating a marker-assisted selection (MAS) approach [12]. Previous studies have revealed the substantial genetic diversity of PH in soybean accessions collected from different geographic regions [20], and many reports indicate significant relationships between various SSRs and PH [27,28,29,30,31,32,33,34,35,36,37,38,39,40,41,42,43,44]. However, comprehensive investigations utilizing SSR markers specifically associated with plant adaptation traits across the world’s soybean collection are still rare. Such studies can potentially identify the diverse germplasm containing favorable alleles for these traits, providing valuable resources for breeders to develop improved cultivars. In this study, we assessed the genetic diversity within the soybean collection using known SSR markers associated with PH to identify the genetic variants that contribute to variations in valuable agronomic traits. The goal of this study was to determine the optimum range of PH for higher productivity and identify SSR markers that directly influence the PH in field conditions in southeastern Kazakhstan.

## 2. Results

### 2.1. Field Assessment of the Studied Collection by Using Main Agronomic Traits

**Figure 1:**
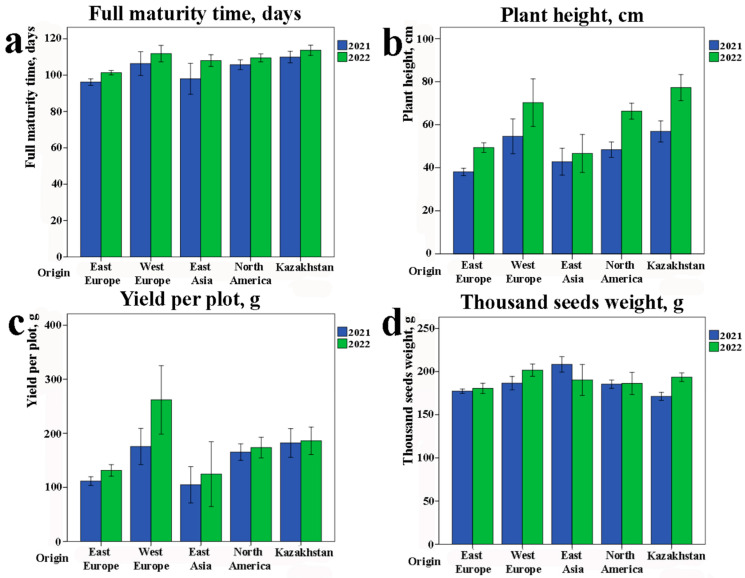
Field performance of soybean accessions according to their origin. Distribution of agronomic traits in the soybean collection by region of origin. Data from two years of field experiments are presented with standard error. (**a**) Full maturity time (VER8); (**b**) plant height (PH); (**c**) yield per plot (YpP); (**d**) thousand-seed weight (TSW).

The study of eleven phenotypic traits demonstrated statistical differences among accessions with different groups of origin (Figure 1). In particular, genotypes from Eastern Europe were the earliest maturing (98.8 ± 1.5 days), while local accessions, on the contrary, were late maturing (111.8 ± 2.9 days) (Figure 1a). The local genotypes also had the highest PH in the world’s collection (Figure 1b). According to yield per plot (YpP), the most productive accessions were from Western Europe (218.7 ± 36.5 g) and Kazakhstan (184.1 ± 18.1 g). The collection was separated into three groups according to their average PH ranges over two years (Table 1). It was revealed that group C (tall PH) showed significantly higher YpP in comparison to group A (short PH, *p* < 0.0001) and group B (middle PH, *p* < 0.0023).

The thousand-seed weight (TSW) was highest in East Asian samples (199.3 ± 10.7 g), but there were no statistically significant differences among the different groups of origin (Figure 1d).

The application of a two-way ANOVA suggested that both environment and genotype heavily influenced key agronomic traits such as VER8, PH, NFN, and NSP. In addition, genotype alone played a vital role in the variation in R2R8 and YpP (Table 2).

**Figure 2:**
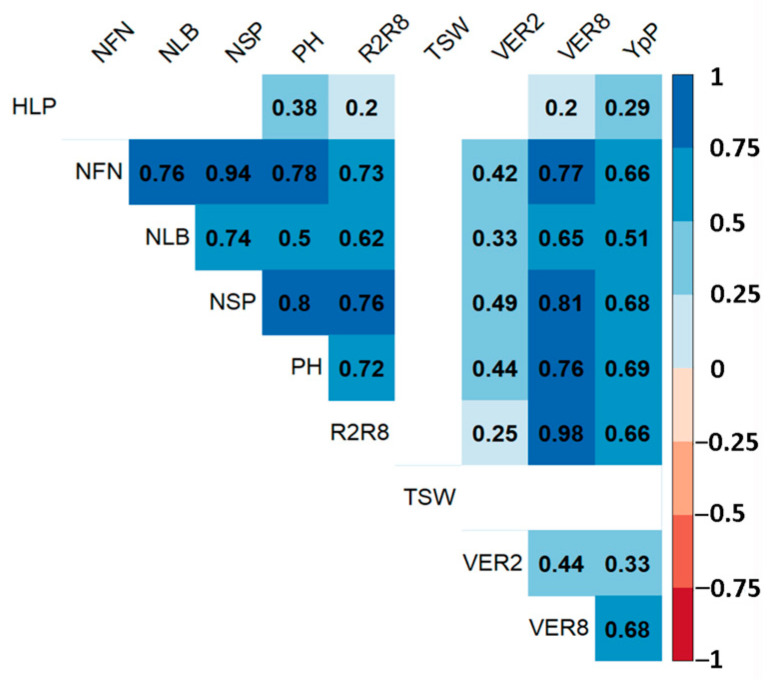
Pearson correlation of agronomic traits of the soybean collection based on two years of average data. Blue cells are positive, and red cells are negative (*p* < 0.01). Blank cells are not significant. VER2—flowering time, days; R2R8—time between flowering and maturity, days; VER8—full maturity time, days; PH—plant height, cm; HLP—the height of the lowest pod, cm; NLB—number of lateral branches, pcs; NFN—number of fertile nodes, pcs; NSP—number of seeds per plant, pcs; TSW—thousand-seed weight, g; YpP—yield per plot, g.

The result of the correlation analysis showed a significant positive relationship (*p* ≤ 0.01) among the studied traits, except for the TSW (Figure 2).

### 2.2. Assessment of the Soybean Collection by Using SSR Markers

The soybean collection was evaluated using 25 SSRs that were previously found to be associated with PH (Table 3 and Table S1). The positions of the twenty-five SSRs in the genome were determined (Table S2, Figure S1); the results suggested that nine SSR markers were in protein-coding regions (Table S2) and sixteen were in intergenic positions. The evaluation of 25 SSRs revealed 109 alleles in the collection, and the average number of alleles per locus was 4.36 (Table 2). The number of alleles per locus ranged from two (Satt428 and Satt600) to eleven (Satt458), and the effective number of alleles ranged from 1.19 (Sat_308) to 4.41 (Sat458), with a mean of 2.32. The mean Nei’s genetic diversity index was 0.546, ranging from 0.152 (Sat_308) to 0.855 (Satt458). The mean polymorphism information content (PIC) value was 0.541 and ranged from 0.140 for Sat_308 to 0.786 for Satt458 (Table 3).

High values of unbiased Nei diversity were observed for all groups of soybean origin except East Asia, which was represented by a small number of accessions. The Fst (fixation index) values confirmed a considerable degree of differentiation among populations in five groups of origin of soybean accessions (Table 4). In addition, applying only five different SSRs (Satt288, Satt371, Satt244, Satt489, and Satt547) appeared to be sufficient to uniquely identify nineteen Kazakhstan soybean accessions, suggesting that SSR markers can be reliable DNA fingerprints of soybean accessions (Figure S2).

The level of genetic diversity in five groups studied with different origins suggested that the value of uh (unbiased Nei’s diversity index) in local accessions is comparable with uh values in samples from other regions. For instance, for samples in Kazakhstan (0.605), this value was less than in Western Europe (0.625) but slightly higher than in Eastern Europe (0.580) and North America (0.583) (Table 4).

### 2.3. Clusterization Analysis of the Studied Collection

**Figure 3:**
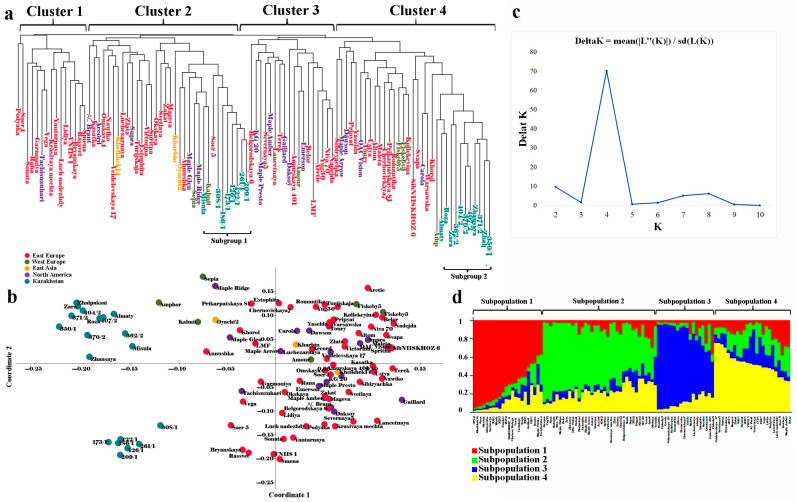
Clusterization of 100 soybean accessions using 25 SSR markers. (**a**) Neighbor-joining tree separating accessions into four clusters; (**b**) PCoA (principal coordinate analysis) confirmed separation of Kazakh samples on the left side of the plot; (**c**) results of the STRUCTURE–HARVESTER method suggested that the optimal number of clusters is four; (**d**) separation of the collection in four clusters using the STRUCTURE package (K4 step).

The clusterization analysis in the population was based on using 25 SSR markers (Table 3). The neighbor-joining tree divided the studied collection into four large clusters (Figure 3a). The local accessions were grouped in Clusters 2 and 4 and formed two separate subgroups: *Subgroup 1* (eight accessions) and *Subgroup 2* (eleven accessions). The principal coordinate analysis (PCoA) also clearly separated local accessions from other genotypes, as they were plotted on the left side of the eigenvalue (Figure 3b).

The separation of samples from Kazakhstan into two different subgroups (Subgroup 1 and Subgroup 2) suggested a drastic difference in the majority of studied agronomic traits. The *t*-test suggested that the highest dissimilarity between the averages of the two groups was in YpP (*p* < 0.0012), followed by VER8, R2R8, and PH (Table 5). The higher values for key traits (R2R4, R2R8, VER8, PH, and YpP) in accessions in Subgroup 2 significantly prevailed over the samples in Subgroup 1 (Table 5). At the same time, TSW did not reveal a big difference between the two subgroups.

### 2.4. The Association of SSR Markers with Main Agronomic Traits

The *t*-test was performed to evaluate the associations of SSR markers with ten studied agronomic traits using the field data for 2021 and 2022 (Table 6). In total, it was found that twelve out of the twenty-five SSRs were significantly associated with at least one studied trait (Table 6). Satt489 appeared to be the only SSR marker associated with a PH-only trait. The remaining five SSRs were also significant for flowering and seed maturation stages (Table 6). The analysis indicated that eight SSRs showed associations with plant adaptation traits (VER2, R2R8, and VER8), nine SSRs with plant morphology (PH, HLP, NLB, and NFN), and eight SSRs with yield components (NSP, TSW, and YpP). The largest number of significant associations, 15 and 16, were found for markers Satt324 and Satt440, respectively. Satt440 showed associations with eight out of ten studied traits, the largest number of associations in this study (Table 6).

Based on the results of the *t*-tests, the six most significant SSR markers with pleiotropic effects were identified: Satt387, Satt324, Satt440, Satt460, Satt244, and Satt288. The phenotypic effects of the associated allele for each of those six markers were calculated (Table S3). In addition, haplotypes for these six markers were generated for accessions of the studied collection, and the total effect of the marker was estimated for each studied trait (Table S4). The cultivars Amour from France and Dawson from the USA had the highest number of associated alleles with agronomic traits in their haplotypes. The cultivars Maple Arrow (Canada), Veidelevskaya 17 (Russia), Sepia (France), Spritna, and Victorina (Ukraine), as well as local cultivars Zara, Almaty, and Zhansaya, appeared to have two alleles with positive effects in their haplotypes (Table S4).

## 3. Discussion

### 3.1. Phenotypic Variation in the Studied Soybean Collection

The collection of 100 soybean accessions was studied for 2 years under field conditions in southeast Kazakhstan. The correlation analysis of field data revealed strong positive relationships among the ten studied agronomic traits, including PH. These positive correlations confirmed previously published associations of yield with PH [45,46,47], number of stems [45], NFN [45,46], and TSW [46,47]. The study of the collection in each environment showed a high variation in analyzed traits, suggesting that the germplasm consists of accessions with diverse origins (Figure 1 and Figure 3). In particular, the field analysis revealed a high potential for samples from Western Europe to breed high-yielding plants in Kazakhstan (Figure 1). Cultivars from Western Europe may be used to expand the genetic diversity of local cultivars. The best-performing cultivars were Amour and Sepia from France. The ANOVA indicated that genotype and environment have significantly affected both plant adaptation and yield-related traits, including PH (Table 2). The comparative assessment of the collection using three groups that were separated according to their PH ranges revealed remarkable differences in yield (Table 1). Particularly, group C (tall PH range) showed significantly higher YpP in comparison to group A (short PH range, *p* < 0.0001) and group B (mid PH range, *p* < 0.0023). Hence, a higher PH is more favorable for soybean productivity in southeast Kazakhstan. The result is in good agreement with previously published reports [5,10,12]. Generally, tall plants may tend to lodge and negatively impact the yield. Particularly, this happens in those soybean-growing regions that have a high precipitation level [5,10,12]. However, in relatively dry conditions in southeast Kazakhstan (Table S5), the range of plants from 80 to 111 cm has provided the best yield performance (Table 1).

### 3.2. Analysis of Population Structure and Polymorphism Level in the Studied Soybean Collection

Diverse soybean collections are essential for preserving and utilizing important genetic resources for breeding cultivars with a high yield [48,49]. In this study, the selected panel consisted of 100 accessions originating in Europe, Asia, North America, and Kazakhstan (Table S6), suggesting a high expected level of genetic diversity. The SSR markers were chosen for this study because previously published reports for various legume crops, including chickpea [50], cluster bean [51], and soybean [52,53], indicated the high informativeness of this class of DNA marker. As expected, the evaluation of the twenty-five SSRs suggested that 14 SSRs with a PIC > 0.5 were considered “highly informative” in the current study; 10 “informative” SSRs had PIC values between 0.5 and 0.25; and only one marker had a PIC ≤ 0.25 as “non-informative” (Table 2), following the classification of Botstein et al. (1980) [54]. The average PIC for the 25 SSRs studied was 0.625, indicating a high polymorphic level. Among twenty-five SSRs, Satt371, Satt243, Satt244, Satt458, and Sat288 were previously successfully used to assess genetic diversity in different soybean collections [55,56,57,58,59]. The results showed that only five SSRs were required to distinguish all nineteen accessions from Kazakhstan (Figure S2, which is comparable with reports using other crops [60]. Overall, this work confirmed a high level of polymorphism in the applied SSRs [61,62,63] and verified their efficiency in the assessment of the genetic diversity of soybeans [61,64,65]. The studied soybean collection demonstrated a certain degree of clear clustering of samples based on their SSR profiles, which was shown using the PCoA plot and NJ tree (Figure 3). For instance, all samples bred in Kazakhstan were grouped on the left side of eigenvalue 1 on the PCoA plot (Figure 3b). Moreover, the NJ tree suggested that 19 Kazakhstan accessions were separated into two subgroups, with Subgroup 1 positioned in Cluster 2 and Subgroup 2 in Cluster 4 (Figure 3a). However, cultivars with other origins were mixed in different subclusters (Figure 3). The poor structuring of samples from other countries possibly reflects the heavy germplasm exchange rate among the breeding communities [66], with a little admixture with germplasm from Kazakhstan.

### 3.3. Association of SSRs with Main Agronomic Traits

The separate evaluation of field data for accessions in Subgroup 1 (eight samples) and Subgroup 2 (eleven samples) formed in the NJ tree (Figure 3a) suggested drastic differences between the two groups in a number of studied traits, including YpP (Table 4). Statistical *t*-test-based differences in YpP between two subgroups mean that the SSRs selected for PH can effectively instrument MAS in southeast Kazakhstan. At the same time, the t-test results indicated differences between groups for R2R4, R2R8, and VER8, indicating the possibility of SSR application in studies of seed maturation time. Satt489 appeared to be the only SSR marker that influenced PH alone; the other five PH-associated SSRs were also significantly associated with flowering time, seed maturation time, and yield components (Table 6).

Correlations between seed yield and other agronomic traits in the current study (Figure 2) led us to suggest the presence of pleiotropic genetic factors. The t-test confirmed this assumption, as seven SSRs were found to be associated with at least two out of the ten studied traits (*p* < 0.05) (Table 6). Four SSRs were associated with only one trait, as is clearly visible in the case of Satt489, which was associated with PH only, and for Satt308 with YpP, Satt600 with HLP, and Satt567 with TSW alone (Table 6). Satt150 and Satt567 were good examples of when SSRs were associated with two or more traits, as they were found to be affected by VER2, R2R8, and VER8 (Table 6). The notable case was Satt567, which was located in the region of the gene *Glyma.07g052300*, which is associated with cytochrome P450. This gene is involved in the biosynthesis of structural polymers, defense against pathogen infection, communication with other organisms, hormonal signaling, herbicide resistance, and stress tolerance [67]. The remaining five SSRs demonstrated pleiotropic effects for several traits (Table 6). Among them, Satt387, previously known to be exceptionally related to plant height and seed yield [29], was also found to be associated with variations in R2R8, NLB, NFN, and NSP (Table 6). Similarly, Satt324 was related to plant height [34,38] and R2R8, VER8, PH, NLB, NSP, YP, YpP, and TSW (Table 6). According to Wang et al. (2019) [68], Satt324 is located in the gene *Glyma.18g065100*, which controls the synthesis of laccase associated with plant defense and stem strength. Another case is Satt440, which was previously found to be associated with plant height, seed weight, and seed yield [29,44,69]. In this study, we reported that Satt440 was involved in the variation in VER2, R2R8, VER8, NLB, NFN, NSP, and YpP (Table 6). Another example of the wide-ranging importance of SSRs is the marker Satt288, which was previously reported to be linked with plant height, seed weight, and seed yield [27,70,71]. This work showed that Satt288 is also associated with R2R8, VER8, NLB, NFN, and NSP (Table 6). Notably, all these multi-traits affecting SSR markers were related to the variation in R2R8. Hence, it can be suggested that these DNA markers may play an important role in the regulation of seed maturation time in soybean. Thus, it is shown that by using informative SSR markers, breeders can accelerate the selection process and improve the efficiency of developing local soybean cultivars with desired agronomic traits. These markers may potentially provide a cost-effective and reliable tool for guiding breeding decisions and enhancing the success rate of soybean improvement programs.

## 4. Materials and Methods

### 4.1. Studied Collection and Field Experiments

The present study aimed to analyze the genetic diversity of 100 soybean accessions from 12 countries. The collection included soybean accessions from 5 distinct geographical regions: Eastern Europe (*n* = 56), Western Europe (*n* = 6), East Asia (*n* = 3), North America (*n* = 16), and Kazakhstan (*n* = 19) (Table S6). Kazakhstan’s part of the collection included both local cultivars and promising lines. The Kazakh Research Institute of Agronomy and Plant Growing (KRIAPI, Almalybak, Almaty region) experimental plots were used for the field experiments in 2021 and 2022. The local cultivar Zhansaya was used as a check cultivar for the Almaty region. Soybean accessions were sown using a nearest-neighbor randomized complete block design (nn-RCBD) with randomly assigned accessions. Each accession was grown in individual 1 m plots (15 cm spaces between neighboring plots) in three replications under watering conditions. The experimental design remained unchanged throughout the two year trials. Ten important agronomic traits of plant adaptation (VER2, R2R8, VER8), morphology (PH, HLP, NLB, NFN), and seed yield components (NSP, TSW, YpP) of soybean were assessed. The field trials were performed according to Korsakov et al. (1968) [72]. Five plants per accession were used for the trait assessment.

### 4.2. DNA Extraction and Genotyping by Using SSR Markers

The DNA was extracted from 4 day old seedlings of soybean accessions in two replicates [73]. The genotyping of the soybean collection was conducted using twenty-five SSR markers (Table S1). These SSRs were selected based on their associations with PH (Table S1). PCR conditions were optimized in order to provide high efficiency and accuracy of amplification [15]. The PCR was performed in a total volume of 20 µL, comprising 20 ng of genomic DNA, 1 U of Taq polymerase, 0.2 mM of each deoxyribonucleotide triphosphate (dNTP), 10 pM of each primer, 1.5 mM of magnesium chloride (MgCl_2_), and a standardized 1× Taq buffer solution. Table S1 summarizes information about the chromosome positions, primers, and motifs of each SSR marker in the analysis.

The PCR products were separated on a QIAxcel Connect System for capillary electrophoresis (QIAGEN, Stockach, Germany) using a QIAxcel DNA High Resolution Kit and QX Alignment Marker (15 bp/3 kb) (Figure S3). The OH500 method was used to run the samples with an injection time of 20 s.

### 4.3. Statistical and Population Analysis

The SPSS 22.0 (https://www.ibm.com/support/pages/spss-statistics-220-available-download (accessed on 14 March 2023)) and STATISTICA 13.2 (TIPCO Software Inc., Palo Alto, CA, USA, https://docs.tibco.com/products/tibco-statistica-13-5-0 (accessed on 14 March 2023)) programs were used for the statistical analysis of phenotyping data. During the analysis, the following statistical terms were estimated: mean value, standard deviation (SD), standard error (SE), correlation, analysis of variance (ANOVA).

The PIC index was calculated according to Botstein et al. (1980) [54]. Based on genetic variability data, markers with PIC > 0.5 were considered highly informative, 0.5 > PIC > 0.25 as informative, and PIC < 0.25 as “non-informative” [54]. To determine the discrimination power of each marker, the number of alleles per locus, the number of effective alleles, the fixation index (Fst), and Nei’s genetic diversity index were calculated using the GenAlex program (ver. 6.5) [74]. To analyze the genetic structure of the studied soybean collection, two methods were used: neighbor-joining (NJ) clustering and principal coordinate analysis (PCoA). Both methods were performed using PAST 3.19 software [75].

Analysis of population structure was performed with the software STRUCTURE (v.2.3.4) using a Bayesian Markov Chain Monte Carlo (MCMC) approach based on mixed and correlated abundance models [76]. The number of hypothesis groups ranging from k = 1 to k = 10 was evaluated using 50,000 burn-in iterations followed by 100,000 recorded iterations. STRUCTURE outputs were analyzed for delta K values (ΔK) with STRUCTURE HARVESTER [77].

A *t*-test was performed using SPSS 22.0 statistical software in order to test associations between 25 SSR markers and studied agronomic traits. The genetic map was drawn using MapChart v.2.3 software [78].

## 5. Conclusions

A diverse collection of soybeans, consisting of 100 accessions, was genotyped using 25 SSR markers that were previously reported to be linked with PH, a key trait for plant adaptation. It was revealed that plants with a tall PH range (80–111 cm) showed significantly higher YpP in comparison to groups with short (20–50 cm, *p* < 0.0001) and middle PH ranges (50–80 cm, *p* < 0.0023). The SSR assessment of the collection showed a high level of variability for the selected SSR markers. In fact, fourteen SSRs were considered highly informative (PIC > 0.5), ten SSRs were relatively informative (*p* = 0.25–0.5), and one SSR was poorly informative (*p* ≤ 0.25). The PCoA plot suggested a clear separation of samples from Kazakhstan from accessions from other regions of the world. The NJ dendrogram has separated nineteen accessions from Kazakhstan into two subgroups in Cluster 2 (eight samples) and Cluster 4 (eleven samples). The application of the t-test suggested that samples in two subgroups of Kazakh soybean were significantly different for VER8, PH, and YpP, confirming the importance of the usage of SSRs in the marker-assisted selection approach. Although 25 markers are not enough for an extensive analysis of breeding collections, at the same time, six SSRs showed a pleiotropic effect and affected multiple agronomic traits (VER2, R2R8, VER8, PH, NFN, NLB, NSP, TSW, and YpP). The haplotypes for these six SSRs were generated for each soybean accession, and their effect was estimated for the studied traits. Thus, evaluated SSR markers can be potentially used as a cost-effective tool in breeding projects to develop new cultivars with higher yield records.

## Figures and Tables

**Table 1:** Yield performance of soybean collection with respect to different plant height (PH) ranges using average data over two years (2020–2021).

PH Groups	n	PH Range, cm	Average PH, cm	Average YpP, g
A	58	20–49.9	39.1 ± 7.2	110.0 ± 6.4
B	31	50–79.9	60.5 ± 6.3	179.3 ± 11.0
C	11	80–111.1	91.5 ± 7.7	248.2 ± 19.4

Note: n—number of accessions; PH—plant height, cm; YpP—yield per plot, g.

**Table 2:** Two-way ANOVA.

Factors	Traits	SS	d.f.	MS	F	P
Year	VER2	272.997	1	272.997	28.666	0.000
	R2R8	72.147	1	72.147	0.623	0.431
	VER8	625.829	1	625.829	4.577	0.034
	PH	3760.536	1	3760.536	12.360	0.001
	HLP	27.930	1	27.930	4.857	0.029
	NLN	23.365	1	23.365	47.827	0.000
	NFN	2171.070	1	2171.070	35.081	0.000
	NSP	19,794.491	1	19,794.491	48.245	0.000
	YpP	15,047.475	1	15,047.475	2.137	0.145
	TSW	437.906	1	437.906	0.434	0.511
Origin	VER2	43.218	4	10.805	1.135	0.342
	R2R8	5435.516	4	1358.879	11.727	0.000
	VER8	6063.577	4	1515.894	11.086	0.000
	PH	18,794.750	4	4698.687	15.444	0.000
	HLP	25.838	4	6.460	1.123	0.347
	NLN	2.907	4	0.727	1.488	0.207
	NFN	1218.281	4	304.570	4.921	0.001
	NSP	11,136.326	4	2784.081	6.786	0.000
	YpP	209,326.569	4	52,331.642	7.432	0.000
	TSW	5080.853	4	1270.213	1.258	0.288
Year xOrigin	VER2	37.244	4	9.311	0.978	0.421
	R2R8	9.090	4	2.272	0.020	0.999
	VER8	65.186	4	16.297	0.119	0.976
	PH	875.544	4	218.886	0.719	0.580
	HLP	18.167	4	4.542	0.790	0.533
	NLN	0.246	4	0.062	0.126	0.973
	NFN	135.675	4	33.919	0.548	0.701
	NSP	932.594	4	233.149	0.568	0.686
	YpP	16,622.417	4	4155.604	0.590	0.670
	TSW	4055.344	4	1013.836	1.004	0.407

Note: SS—the sum of squares due to the source; d.f.—degrees of freedom; MS—the mean sum of squares due to the source; VER2—flowering time, days; R2R8—time between flowering and maturity, days; VER8—full maturity time, days; PH—plant height, cm; HLP—the height of the lowest pod, cm; NLB—number of lateral branches, pcs; NFN—number of fertile nodes, pcs; NSP—number of seeds per plant, pcs; TSW—thousand-seed weight, g; YpP—yield per plot, g.

**Table 3:** Assessment of the level of genetic diversity of SSR loci associated with plant height.

SSR Loci	Chr	na	ne	Ho	uHe	uh	PIC
Satt428	2	4	2.45	0.128	0.531	0.550	0.539
Satt600	2	5	1.57	0.061	0.346	0.357	0.340
Satt387	3	2	1.50	0.000	0.318	0.333	0.322
Satt307	6	6	2.49	0.065	0.538	0.582	0.731
Satt371	6	6	3.13	0.134	0.663	0.684	0.703
Satt460	6	3	1.43	0.011	0.281	0.295	0.290
Satt489	6	5	3.19	0.000	0.600	0.626	0.684
Satt557	6	4	2.25	0.000	0.497	0.526	0.563
Satt150	7	3	1.66	0.000	0.414	0.453	0.319
Satt308	7	5	2.35	0.064	0.590	0.634	0.660
Satt567	7	5	2.85	0.000	0.683	0.747	0.661
Satt153	10	5	2.02	0.046	0.448	0.465	0.455
Satt243	10	5	2.79	0.061	0.650	0.699	0.675
Satt197	11	4	2.08	0.032	0.527	0.572	0.493
Satt509	11	3	1.70	0.075	0.378	0.391	0.423
Satt335	13	3	1.75	0.025	0.437	0.479	0.357
Satt263	15	4	2.32	0.046	0.585	0.630	0.598
Satt244	16	7	2.77	0.048	0.642	0.689	0.732
Satt547	16	4	2.47	0.063	0.532	0.551	0.642
Sat_308	18	3	1.19	0.007	0.148	0.152	0.140
Satt288	18	8	2.35	0.198	0.557	0.567	0.624
Satt309	18	3	1.57	0.033	0.336	0.347	0.338
Satt324	18	5	2.51	0.101	0.591	0.619	0.667
Satt440	20	4	2.73	0.000	0.560	0.584	0.644
Sct189	20	3	2.19	0.060	0.565	0.608	0.499
Mean value		4.36	2.21	0.050	0.497	0.526	0.524
Standard error		0.33	0.33	0.007	0.019	0.021	0.028

Note: Chr—chromosome; na—number of alleles per locus; ne—number of effective alleles; Ho—observed heterozygosity; uHe—unbiased expected heterozygosity; uh—unbiased Nei diversity; PIC—polymorphism information content.

**Table 4:** Genetic diversity in five groups of soybean origin based on SSR markers.

Region	ne	Ho	He	uHe	Fst	uh
Eastern Europe	2.60	0.078	0.567	0.573	0.854	0.580
Western Europe	2.23	0.048	0.504	0.551	0.894	0.625
East Asia	1.44	0.040	0.229	0.275	0.764	0.240
North America	2.51	0.045	0.546	0.564	0.929	0.583
Kazakhstan	2.29	0.040	0.507	0.521	0.921	0.605
Mean value	2.21	0.050	0.471	0.497	0.884	0.527
Standard error	0.08	0.007	0.020	0.020	0.019	0.024

Note: na—number of alleles per locus; ne—number of effective alleles; Ho—observed heterozygosity; uHe—unbiased expected heterozygosity; Fst—fixation index; uh—unbiased Nei diversity.

**Table 5:** The averaged data for ten agronomic traits in two subgroups of Kazakhstan accessions.

Subgroups	N	Year	VER2	R2R8	VER8	PH	HLP	NLB	NFN	NSP	TSW	YpP
Subgroup 1	8	2021	31.75	68.75	100.50	44.45	7.75	0.38	11.63	24.99	170.38	105.41
		2022	34.63	69.75	104.38	58.69	7.54	1.80	23.02	60.00	194.63	130.46
		Mean	33.19	69.25	102.44	51.57	7.64	1.09	17.32	42.49	182.50	117.94
		SE	1.72	0.22	1.93	6.33	0.24	0.76	5.57	15.80	11.57	1.18
Subgroup 2	11	2021	34.55	82.27	116.82	65.94	7.15	0.74	15.08	42.29	171.82	237.95
		2022	36.36	84.09	120.45	90.75	11.27	1.80	27.68	78.69	192.73	226.55
		Mean	35.45	83.18	118.64	78.34	9.21	1.27	21.38	60.49	182.27	232.25
		SE	0.80	1.04	1.84	12.43	1.96	0.52	6.33	19.14	10.92	3.82
*p*-value for 2 subgroups			0.158	0.002	0.001	0.007	0.152	0.568	0.187	0.063	0.977	0.001

Note: N—number of accessions; VER2—flowering time, days; R2R8—time between flowering and maturity, days; VER8—full maturity time, days; PH—plant height, cm; HLP—the height of the lowest pod, cm; NLB—number of lateral branches, pcs; NFN—number of fertile nodes, pcs; NSP—number of seeds per plant, pcs; TSW—thousand-seed weight, g; YpP—yield per plot, g.

**Table 6:** The association of SSR markers with the studied agronomic traits based on *t*-tests. The output of the *t*-test is given as a *p*-value.

SSR Markers	Chr	VER2	R2R8	VER8	PH	HLP	NLB	NFN	NSP	TSW	YpP
Satt387	2	ns	0.045 ^2^	ns	0.018 ^1^	ns	0.018 ^1^ 0.004 ^2^ 0.008 ^3^	0.001 ^1^	0.002 ^1^ 0.007 ^3^	ns	0.013 ^2^ 0.002 ^3^
Satt489	3	ns	ns	ns	0.007 ^2^ 0.034 ^3^	ns	ns	ns	ns	ns	ns
Satt557	4	ns	ns	ns	ns	ns	ns	ns	ns	0.031 ^1^	ns
Satt150	4	ns	0.044 ^2^	0.031 ^2^	ns	ns	ns	ns	ns	ns	ns
Satt567	6	0.040 ^1^	ns	ns	ns	ns	ns	ns	ns	ns	ns
Satt324	6	Ns	0.038 ^1^ 0.016 ^2^ 0.021 ^3^	0.022 ^1^ 0.017 ^2^ 0.016 ^3^	0.020 ^1^ 0.027 ^2^ 0.016 ^3^	ns	0.036 ^2^	ns	0.030 ^1^ 0.028 ^2^ 0.016 ^3^	0.036 ^2^ 0.023 ^3^	0.048 ^1^ 0.043 ^2^ 0.014 ^3^
Satt440	6	0.000 ^1^ 0.000 ^3^	0.002 ^1^ 0.002 ^2^ 0.001 ^3^	0.000 ^1^ 0.000 ^2^ 0.000 ^3^	0.002 ^1^ 0.006 ^3^	ns	0.000 ^1^ 0.004 ^3^	0.000 ^1^ 0.036 ^3^	0.000 ^1^ 0.019 ^3^	ns	0.001 ^1^ 0.042 ^2^ 0.002 ^3^
Satt600	6	ns	ns	ns	ns	0.030 ^2^	ns	ns	ns	ns	ns
Satt460	7	ns	0.038 ^1^ 0.042 ^3^	ns	0.039 ^1^ 0.041^3^	ns	ns	ns	ns	ns	0.024 ^2^ 0.046 ^3^
Satt244	10	ns	0.006 ^1^ 0.006 ^2^ 0.004 ^3^	0.019 ^1^ 0.019 ^2^ 0.016 ^3^	ns	ns	0.026 ^1^	ns	ns	ns	0.013 ^1^
Satt288	11	ns	0.031 ^1^ 0.004 ^2^ 0.010 ^3^	0.003 ^1^ 0.011 ^2^ 0.016 ^3^	0.002 ^1^ 0.000 ^2^ 0.000 ^3^	ns	0.067 ^1^	0.005 ^1^ 0.043 ^2^ 0.014 ^3^	0.001 ^1^ 0.021 ^2^ 0.004 ^3^	ns	0.000 ^2^ 0.000 ^3^
Satt308	17	ns	ns	ns	ns	ns	ns	ns	ns	ns	0.029 ^2^

Note: Chr—chromosome: ^1^ *p*-value for 2021, ^2^ *p*-value for 2022, ^3^ *p*-value for average data, ns—not significant; VER2—flowering time, days; R2R8—time between flowering and maturity, days; VER8—full maturity time, days; PH—plant height, cm; HLP—the height of the lowest pod, cm; NLB—number of lateral branches, pcs; NFN—number of fertile nodes, pcs; NSP—number of seeds per plant, pcs; TSW—thousand-seed weight, g; YpP—yield per plot, g.

## Data Availability

All data are available in the article and Supplemental Files.

## Supplementary Materials

The following supporting information can be downloaded at: https://www.mdpi.com/article/10.3390/plants12193445/s1, Figure S1: Localization of 25 SSR markers associated with plant height. The SSR markers used in this study and QTLs from Soybase.org associated with plant height are marked in blue. Known genes previously reported to control plant height are marked in red; Figure S2: Identification steps for 19 Kazakhstan soybean accessions based on analysis of SSRs; Figure S3: Fragment of an electropherogram of PCR amplification products obtained with primer Satt440; Table S1: The list of simple sequence repeat (SSR) markers associated with plant height (PH), arranged according to their chromosomal positions; Table S2: Physical positions of used in this study 25SSR markers in the soybean genome; Table S3: Effect of genotypes based on associated alleles on 10 studied agronomic traits; Table S4: Haplotype of the studied collection based on the six SSR markers and their total phenotypic effect on the main agronomic traits; Table S5: Metrological data of the experimental site for 2021–2022; Table S6: List of accessions and distribution by group of origin.
