# Supplementary figures and images for: Genetic Diversity Analysis of Soybean Collection Using Simple Sequence Repeat Markers
Source: Plants (Basel). 2023 Sep 30;12(19):3445. doi: 10.3390/plants12193445 (PMC10575313; doi:10.3390/plants12193445)

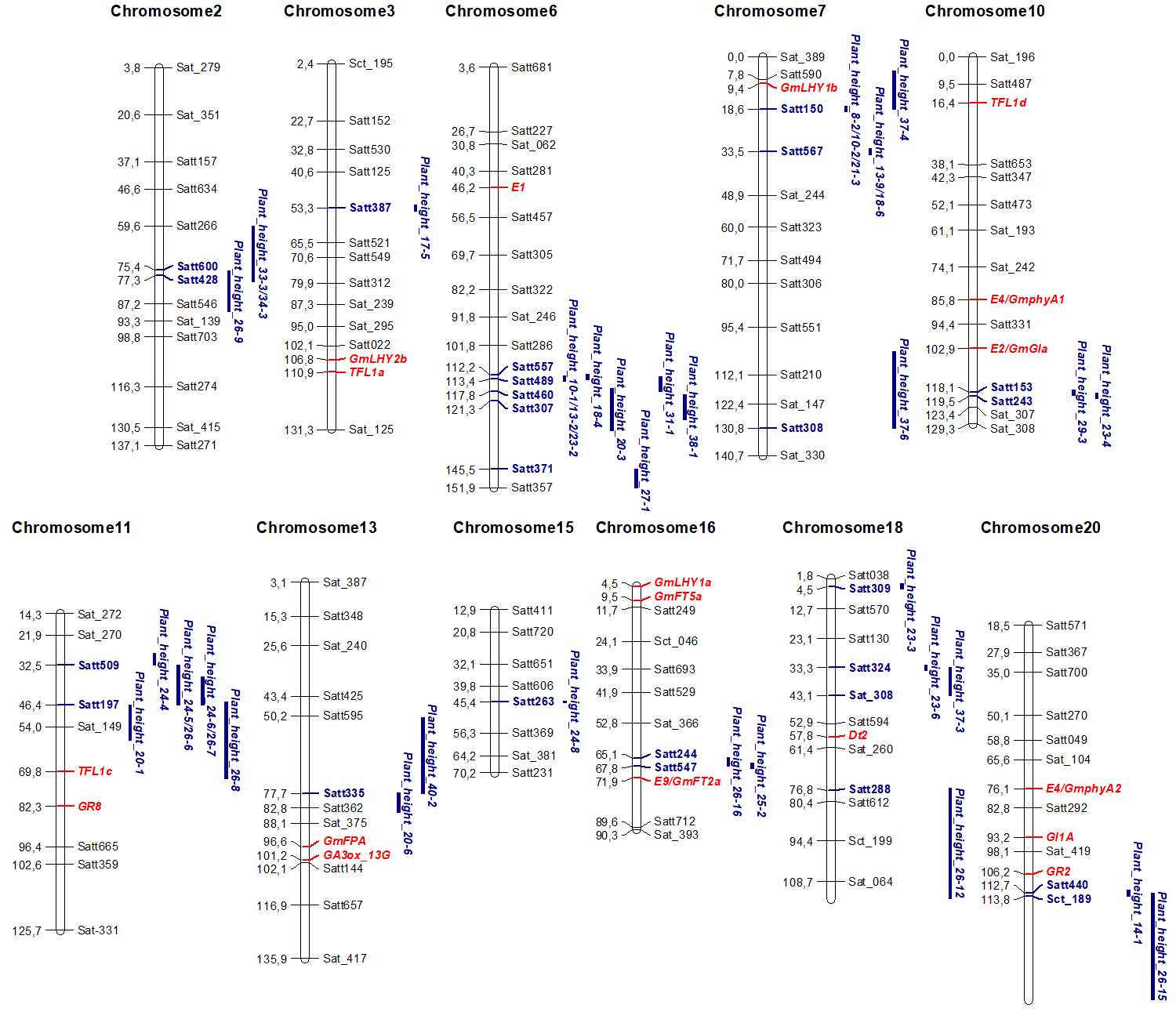

Supplement: Supplementary file 1 [file plants-12-03445-s001.zip › Figure S1. Localization of 25 SSR markers associated with plant height.png]

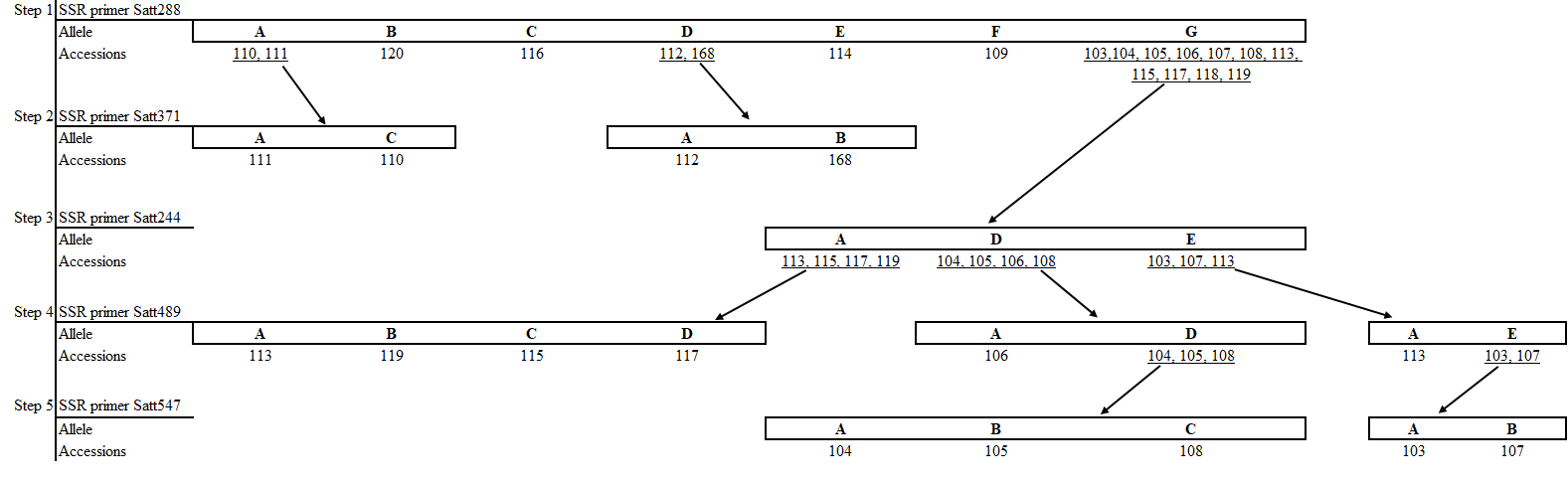

Supplement: Supplementary file 1 [file plants-12-03445-s001.zip › Figure S2. Identification steps for 19 Kazakhstan soybean accessions based on analysis of SSRs.png]

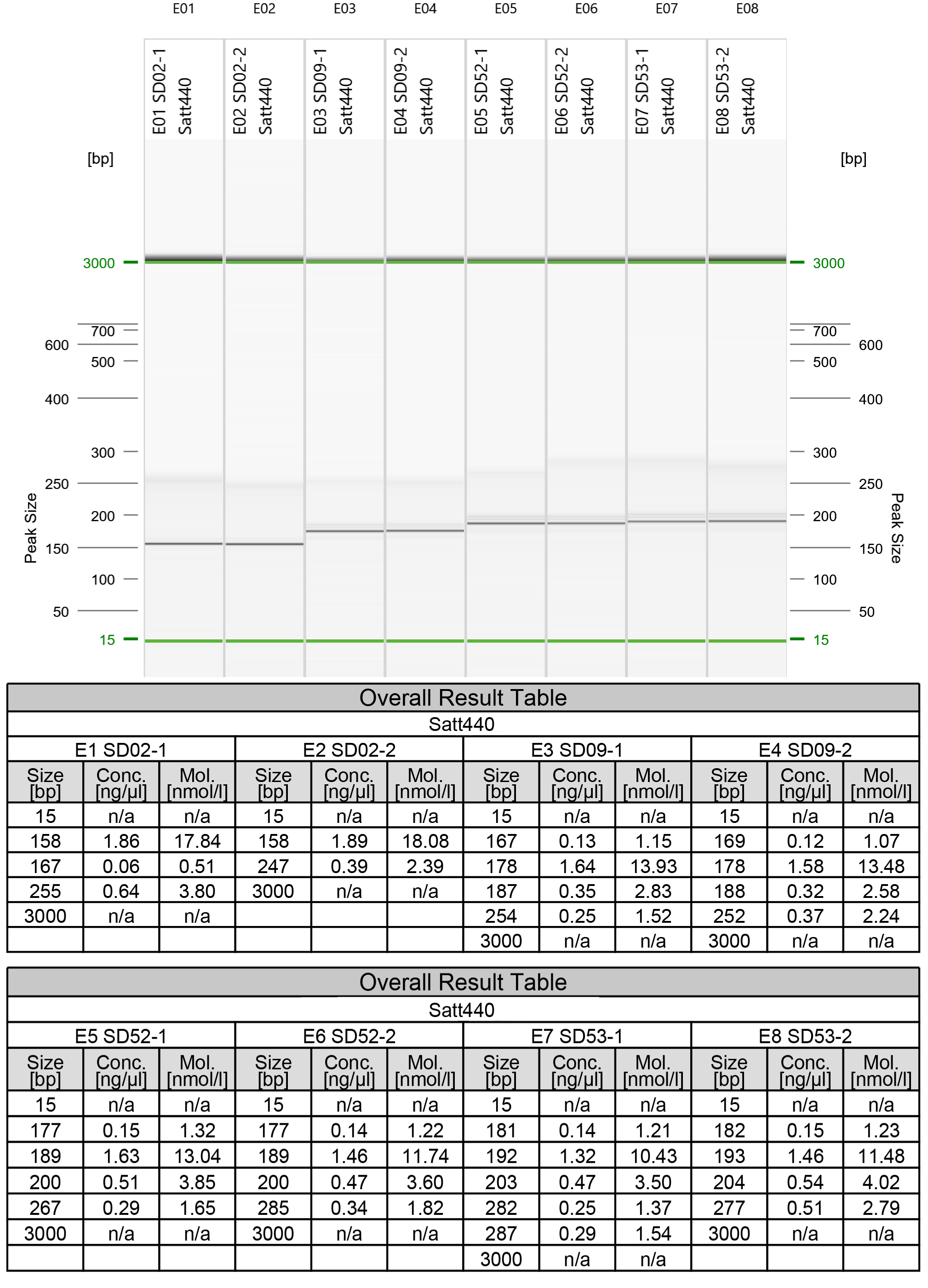

Supplement: Supplementary file 1 [file plants-12-03445-s001.zip › Figure S3. Fragment of electropherogram of PCR amplification products obtained with primer Satt440.png]
